# Dynamics of cheater invasion in a cooperating population of *Pseudomonas aeruginosa*

**DOI:** 10.1038/s41598-019-46651-5

**Published:** 2019-07-15

**Authors:** Xiaoyin Feng, Maxim Kostylev, Ajai A. Dandekar, E. Peter Greenberg

**Affiliations:** 1 Guangdong Innovative and Entrepreneurial Research Team of Sociomicrobiology Basic Science and Frontier Technology, South China Agricultural University, Guangzhou, 510642, People’s Republic of China; 2 Guangdong Province Key Laboratory of Microbial Signals and Disease Control, Department of Plant Pathology, South China Agricultural University, Guangzhou, 510642 People’s Republic of China; 3 Department of Microbiology, University of Washington, Seattle, WA 98195 USA; 4 Department of Medicine, University of Washington, Seattle, WA 98195 USA

**Keywords:** Microbiology, Bacteria

## Abstract

*Pseudomonas aeruginosa* quorum sensing (QS) regulates expression of dozens of genes in a cell density-dependent manner. Many QS-regulated genes code for production of extracellular factors, “public goods” that can benefit the entire population. This cooperation encourages individuals to cheat by using but not producing public goods. QS also controls expression of a limited number of genes encoding “private” cellular enzymes like Nuh, an enzyme involved in adenosine catabolism. Growth of *P*. *aeruginosa* on casein requires QS-regulated production of an extracellular protease and is an example of cooperative behavior. When *P*. *aeruginosa* is transferred daily on casein, QS mutants emerge. These cheaters have mutations in *lasR*, which encodes the primary QS transcription factor. When growth is on casein and adenosine, cheater emergence is constrained. Here, we report the dynamics of LasR mutant invasion during growth on casein or casein plus adenosine. We show that LasR mutants have the greatest advantage during early to mid-logarithmic growth on casein. Addition of adenosine to casein medium constrains cheaters throughout growth. Our data support the view that co-regulation of the public protease and the private nucleosidase by QS stabilizes cooperation, and the data are not consistent with other proposed alternate hypotheses.

## Introduction

*Pseudomonas aeruginosa* uses a complex quorum sensing (QS) system to activate dozens of genes in a cell density-dependent manner^1,2^. *P*. *aeruginosa* has two complete acyl-homoserine lactone (acyl-HSL) QS circuits, LasR-LasI and RhlR-RhlI (for reviews, see refs^3–5^). LasI catalyzes synthesis of the diffusible signal *N*-3-oxododecanoyl-HSL (3OC12-HSL), which binds to and activates the transcriptional regulator LasR. Similarly, RhlI catalyzes the synthesis of *N*-butyryl-HSL (C4-HSL), which activates the transcriptional regulator RhlR. In well-studied laboratory strains of *P*. *aeruginosa*, such as PAO1, the two QS circuits are arranged hierarchically, such that LasR activates the expression of *rhlR-I*. As a result, LasR-null mutants of PAO1 do not exhibit QS-regulated phenotypes, including those that are regulated by RhlR. LasR also positively regulates a non-acyl-HSL QS circuit, mediated by the *Pseudomonas* quinolone signal (PQS) and its biosynthetic precursor 2-heptyl-4-quinolone (HHQ)^6–9^. Together, these three QS circuits induce the production of many extracellular products, including toxins, surfactants, and enzymes^10^.

Extracellular products can benefit the entire population of cells and are a type of “public good”. By extension, because QS regulates the production of public goods, it also regulates cooperative behavior^11^. In the context of evolution, maintenance of cooperation presents a social dilemma^11–13^. Since the production of public goods has a metabolic cost for individual cells, there is an incentive for individual cells to “cheat” by not producing public goods or by producing less than a fair share of the goods. These cells gain an advantage because they continue to reap the benefit of public-goods production by cooperators. The emergence of cheaters in *P*. *aeruginosa* populations has been demonstrated *in vitro*^14–16^. Growth of *P*. *aeruginosa* on casein or bovine serum albumin (BSA) as the sole carbon and energy source requires secretion of the QS-regulated protease elastase. When *P*. *aeruginosa* is grown by daily passage under these conditions, QS-deficient LasR mutants emerge within 10–14 days, and their frequency usually stabilizes at around 30–50% of the population^14–17^. These mutants have a negative frequency-dependent fitness advantage, as they grow best when they are rare in a population of cooperators^14,15^.

*P*. *aeruginosa* has evolved mechanisms that enhance QS-regulated cooperative behaviors^18^. One such mechanism is the acquisition of loss-of-function mutations in the non-QS-regulated gene *psdR*, which encodes a transcriptional repressor of genes that are important for the uptake and metabolism of dipeptides^19^. When *P*. *aeruginosa* PAO1 is cultured on casein as the sole carbon source, PsdR mutants emerge within days and these mutants dominate the population. *psdR* mutations increase the fitness of the population and enable it to tolerate a higher frequency of cheaters without population collapse^20^. PsdR mutants grow more rapidly than wild-type (WT) on casein, and it is unclear whether the timing of serial transfers in published evolution studies (usually 24 h) of *P*. *aeruginosa* could introduce an unexpected bias.

One mechanism described to control cheating in *P*. *aeruginosa* is QS regulation of the nucleoside hydrolase Nuh, a cellular enzyme^16^. Nuh is involved in catabolism of adenosine and inosine^21–24^ for use as a carbon and energy source. Unlike secreted products, which benefit the whole population, Nuh is a “private good” that is not shared. QS regulation of *nuh* expression provides an incentive to maintain cooperation when adenosine is present during growth. When *P*. *aeruginosa* is grown on casein and adenosine as sole carbon and energy sources, the frequency of LasR mutants is much lower than that on casein alone^16^.

The hypothesis that QS regulation of *nuh* directly restrains cheater emergence in populations cooperating to utilize casein with adenosine has been questioned^25^. A possible alternative explanation is that when *P*. *aeruginosa* is passaged in the presence of abundant adenosine with casein, the cells quickly adapt to preferentially utilize adenosine instead of casein as a carbon source. In this hypothesis, the adaptation to adenosine is non-social, thus eliminating the incentive for LasR cheaters to arise^25^.

Although the emergence of *P*. *aeruginosa* LasR mutants during evolution on casein is well established, the dynamics of cheater invasion have not been carefully studied. We are interested in understanding when during growth LasR mutants have the greatest fitness advantage and whether this advantage is altered by the loss of PsdR. We also wanted to address the question of how the addition of adenosine to casein-containing medium affects cheater invasion dynamics. Thus, we performed time-course growth experiments that examine in detail the ability of a LasR mutant to invade a cooperating population growing in minimal medium containing casein with or without adenosine.

## Results

### Fitness advantage of LasR mutant during growth with parent

**Figure 1:**
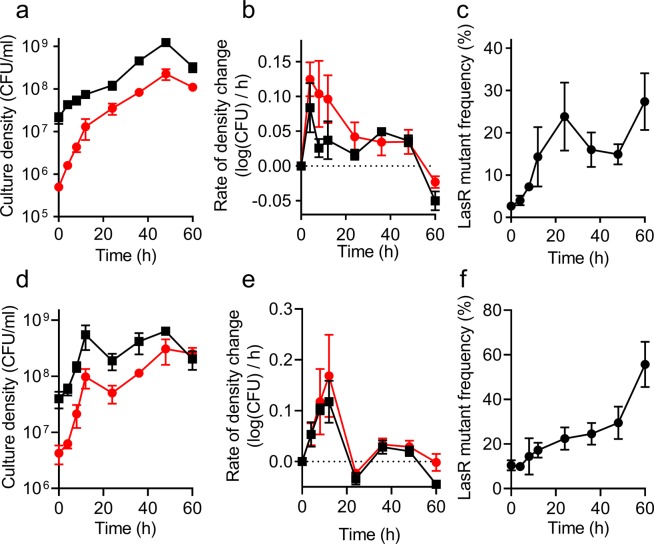
Co-cultures of WT PAO1 (squares) and LasR mutant (circles) in 1% casein broth. Initial frequency of the LasR mutant was 1% (**a**–**c**) or 10% (**d**–**f**). (**a**,**d**) Cell density was measured as CFU (see Methods). (**b**,**e**) Rate of change in cell density, calculated from data in panels a,d, respectively (see Methods). (**c**,**f**) LasR mutant frequency, calculated from data in panels a and d, respectively. In panel b, the rate of change of the LasR variant is significantly higher than that of the WT between 0–24 h (p = 0.03 by *t*-test with correction for multiple comparisons). Beyond 24 h, there is no significant difference. The rates of change of WT and the LasR variant were similar throughout in panel e. All data are means of three replicates and the error bars represent the SEM.

In experiments where *P*. *aeruginosa* is grown on casein as the sole carbon and energy source for 24 h, LasR mutants have a fitness advantage over wildtype (WT) cells^14,16,20^. To determine when during growth this advantage is most prominent, we co-cultured LasR mutant and WT PAO1 in a minimal medium containing casein as the sole carbon and energy source (casein broth). We inoculated the mutant at initial frequencies of 1% or 10% and monitored growth of both strains for 60 h (Fig. 1). The LasR mutant contained a gentamicin resistance marker at a neutral chromosomal site so that we could distinguish it from PAO1 by selective plating. The LasR mutant had the greatest advantage over WT during the initial 24 h (early to mid-logarithmic phases) (Fig. 1a,b,d,e). This was reflected in the change of the LasR mutant frequency, which increased from 1% initial frequency to 24% in 24 h (Fig. 1c) and from 10% initial frequency to 17% in 12 h (Fig. 1f). The fitness advantage of the LasR mutants was greater when its inoculation frequency was 1% compared to initial frequency of 10%, as evidenced by the relative increase of the mutant growth rate over WT (Fig. 1b,e). This observation is consistent with previous end-point experiments showing that LasR mutants have a negative frequency-dependent fitness advantage during growth on casein or BSA^14,15^. We did not observe a clear fitness advantage for either strain during late logarithmic and early stationary phases, indicating that invasion by cheaters is not continuous over the course of an experiment. We presume cheater invasion slows in late logarithmic growth as a result of negative frequency dependence. After 60 h, the proportion of LasR mutants again began to increase rapidly, reaching 27% and 56% in cultures initiated at 1% and 10% mutant frequencies, respectively. This increase resulted primarily from a greater rate of WT cell death, as evidenced by a decrease of WT cell density (Fig. 1a,d). After 60 h of growth, the pH of the cultures had increased to 8.7 from 6.7 at the time of inoculation. This difference in the death rate between the two strains in late stationary phase is consistent with a prior report that LasR mutants are more resistant than WT to alkaline stress^24^. Together, these results highlight that differences in mutant frequencies in various studies might reflect different sampling endpoints.

### Deletion of *psdR* does not affect the dynamics of invasion by the LasR mutant

**Figure 2:**
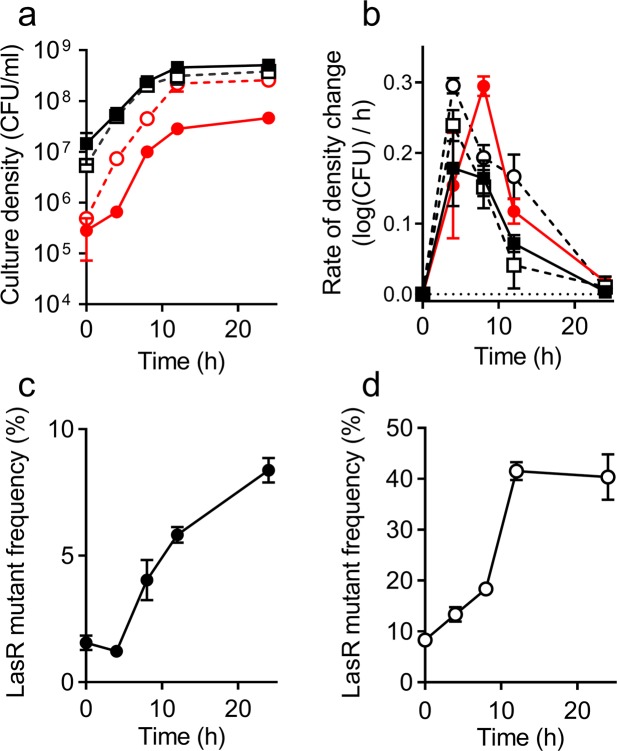
Co-cultures of PAO1 PsdR mutant (squares) and PsdR-LasR double mutant (circles) in 1% casein broth. Initial frequency of the LasR mutant was 1% (filled symbols) or 10% (open symbols). (**a**) Cell density was measured as CFU (see Methods). (**b**) Rate of change in cell density, calculated from the data in panel a (see Methods). (**c**,**d**) PsdR-LasR mutant frequency, calculated from data in panel a. All data are means of three replicates and the error bars represent the SEM of the data.

Loss-of-function mutations in *psdR* have been shown to increase the fitness of both WT and LasR mutant *P*. *aeruginosa* growing on casein^20^. PsdR mutants are susceptible to invasion by PsdR-null, LasR-null cheaters, but not by LasR mutants without a *psdR* mutation^20^. To examine the effect of *psdR* deletion on dynamics of cheater invasion, we co-cultured a PsdR mutant with a PsdR-LasR double mutant in casein broth. We inoculated the PsdR-LasR double mutants at initial frequencies of 1% or 10% and monitored growth throughout a 24 h period (Fig. 2). As expected, deletion of *psdR* increased the growth rate of both cooperators and cheaters (Fig. 2a,b). The observed peak growth rate of the PsdR mutant strains was about twice that of WT PAO1 and the LasR mutant (Fig. 2b; compare to Fig. 1b,e). However, the general pattern of invasion by the LasR mutant was not substantially altered when both cooperators and cheaters carried a *psdR* deletion. As observed with WT PAO1 (Fig. 1), the fitness advantage of the LasR mutant was most pronounced during log-phase growth (Fig. 2).

### LasR mutants do not invade the population in casein broth with adenosine

**Figure 3:**
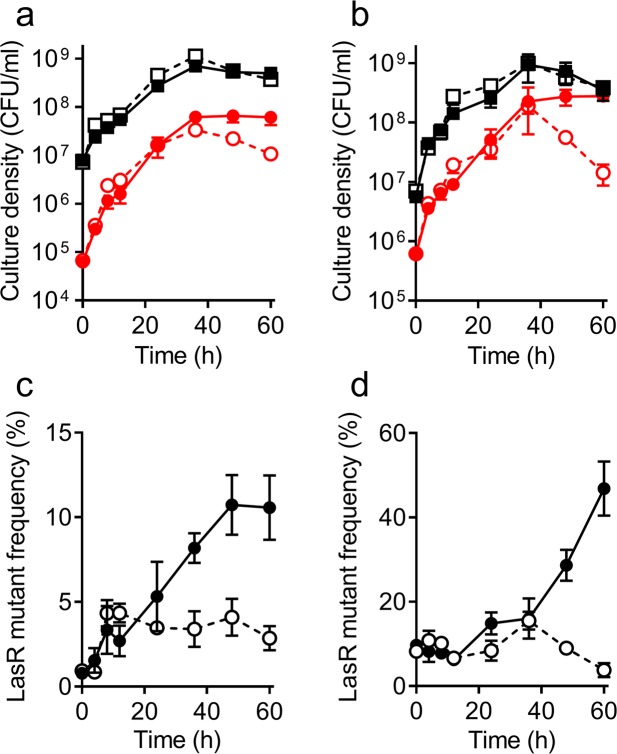
Co-cultures of WT PAO1 (squares) and LasR mutant (circles) in 0.75% casein (solid lines) and 0.75% casein plus 0.75% adenosine (dotted lines) broth. Initial frequency of the LasR mutant was 1% (**a**,**c**) or 10% (**b**,**d**). (**a**,**b**) Cell density, determined as CFU (see Methods). (**c**,**d**) LasR mutant frequency, calculated from data in panels a and b, respectively. All data are means of three replicates and the error bars represent the SEM of the data.

Many genes that are regulated via QS encode secreted enzymes or enzymes that make secreted products^1,26^, which together can benefit the entire population of cells. However, a subset of QS-regulated genes, including *nuh*, encode cellular enzymes that benefit individual cells. We have reported that the co-regulation of cellular functions with public-goods production can provide an incentive to maintain QS gene regulation within a population by increasing the fitness of cooperating cells. When *P*. *aeruginosa* was grown in a minimal medium containing both casein and adenosine, the emergence of LasR mutants was constrained, in contrast to growth on casein as the sole carbon source^16^. Our interpretation of these experiments has been questioned^25^, with a proposed alternate explanation of selective use of adenosine instead of casein as a carbon and energy source. Therefore, we examined in detail how the addition of adenosine to casein broth affected the ability of a LasR mutant to invade a cooperating population. We co-cultured the LasR mutant and WT PAO1 in 0.75% casein broth or 0.75% casein plus 0.75% adenosine broth, again with initial LasR mutant frequencies of 1% or 10% (Fig. 3). We chose these conditions because we previously observed that the addition of at least 0.75% adenosine was sufficient to restrict cheater emergence during *P*. *aeruginosa* growth in casein broth^16^. Consistent with our prior report, addition of adenosine to the casein broth prevented LasR mutants from invading the population from either a 1% or 10% initial frequency (Fig. 3c,d). The constraint of LasR mutants was most evident in stationary phase, where CFU of LasR mutants decreased more rapidly than those of WT (Fig. 3a,b). Although the presence of adenosine in the medium increased the rate of LasR mutant cell death, this effect could not be attributed to the pH, which was 8.8 after 60 h. This result is inconsistent with the idea that LasR mutants do not emerge because cells preferentially use adenosine over casein during most of logarithmic growth.

### Constraint of LasR mutant infiltration in the presence of adenosine is stronger in an adenosine-adapted population of PAO1

When *P*. *aeruginosa* grows on adenosine as a carbon and energy source, it adapts to grow significantly faster than the WT on pure adenosine through gene duplication-amplification events^27^. In an earlier study, selected isolates that had evolved in 0.9% adenosine plus 0.1% casein had doubling times on adenosine in the range of 5–12 h, well below the 45 h doubling time of WT PAO1 on adenosine alone^27^. These adapted isolates all contained multiple copies of *nuh* and several neighboring genes^27^. We asked whether the gene-amplification adaptation of *P*. *aeruginosa* to adenosine would affect the dynamics of LasR mutant invasion of a cooperating population grown in casein plus adenosine broth. Specifically, we wanted to examine the possibility that the primary advantage of adapted strains over QS-null cheaters could be explained by preferential utilization of adenosine over casein during early logarithmic growth.

**Figure 4:**
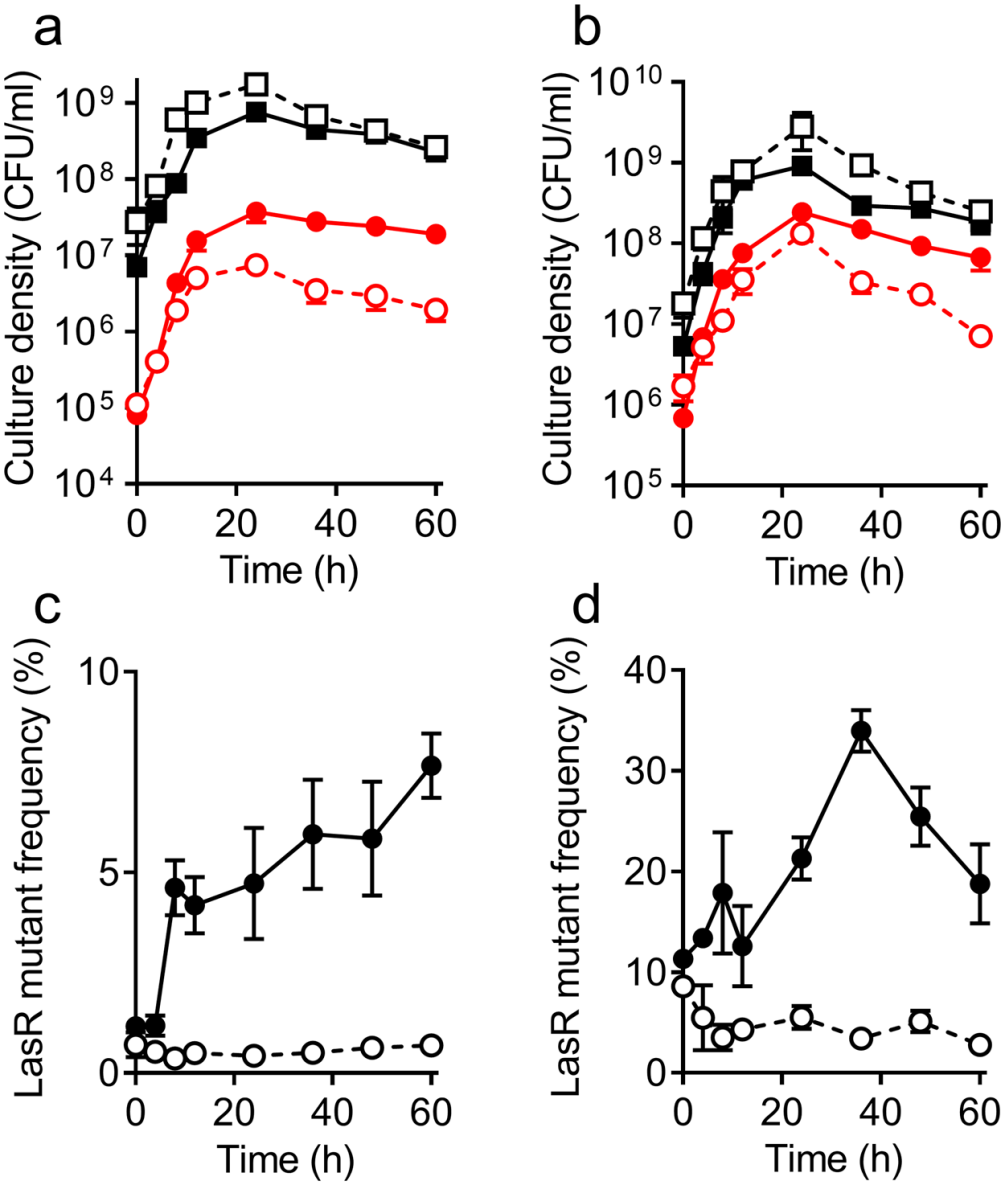
Co-cultures of adenosine-adapted variant D (squares) and a LasR-null mutant of variant D (circles) in 0.75% casein (solid lines) or 0.75% casein plus 0.75% adenosine (dotted lines) broth. Initial frequency of the LasR mutant was 1% (**a**,**c**) or 10% (**b**,**d**). (**a**,**b**) Cell density, determined as CFU (see Methods). (**c**,**d**) LasR mutant frequency, calculated from data in panels a and b, respectively. All data are means of three replicates and the error bars represent the SEM of the data.

We selected an adenosine-adapted isolate of strain PAO1 from our earlier study, which had an adenosine growth doubling time of 5 h (called “variant D” in ref.^27^). This variant has 10 copies of a 12.4 kb genomic region, which includes the genes PA0143 (*nuh*) to PA0148 (a gene encoding adenine deaminase). We deleted *lasR* from this isolate and co-cultured the parent and LasR mutant strains in either 0.75% casein alone or 0.75% casein plus 0.75% adenosine (Fig. 4). In casein alone, the LasR mutant of the adenosine-adapted variant was able to invade the population of parent cooperating cells in a similar manner as that observed in a WT PAO1 background (Fig. 4c,d; compare to Fig. 1). On the other hand, in casein plus adenosine broth, the frequency of the LasR mutant remained low throughout growth, regardless of the initial mutant frequency (Fig. 4c,d). Furthermore, addition of adenosine to casein resulted in lower colony counts of LasR mutants after 8 h of growth and beyond (Fig. 4a,b). This observation contrasts with co-cultures of non-adapted PAO1-derived strains, in which LasR mutant CFU were similar with or without adenosine during early stages of growth but decreased rapidly in the late stationary phase when adenosine was present (Fig. 3a,c).

**Figure 5:**
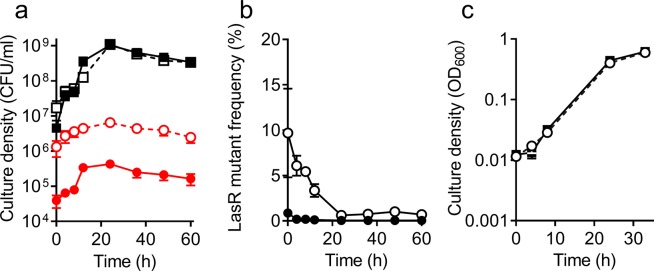
Co-cultures of WT variant D (squares) and LasR-null mutants of variant D (circles) in 0.75% adenosine broth. Initial frequency of the LasR mutant was 1% (filled symbols) or 10% (open symbols). (**a**) Cell density, determined as CFU (see Methods). (**b**) LasR mutant frequency, calculated from data in panel a. (**c**) Growth curves of adenosine-evolved variant D (filled squares) and a LasR mutant of variant D (open circles) in 0.75% adenosine. OD_600_ was measured in a 96 well plate, using a microplate reader. All data are means of three replicates and the error bars represent the SEM of the data.

We also analyzed the dynamics of LasR mutant invasion in co-culture with the parent strain (variant D) on pure adenosine (Fig. 5). Consistent with results of the casein plus adenosine experiment, the frequency of the LasR mutant immediately decreased after inoculation and remained low during all stages of growth (Fig. 5b). On the other hand, we did not observe growth differences of the variant WT and LasR mutant pure cultures with adenosine as the sole carbon source (Fig. 5c). We attribute the faster growth of the LasR mutant in adenosine to the extra copies of *nuh* and the adenine deaminase-encoding gene PA0148 that are present in this variant, and which have been shown to confer rapid growth on adenosine^27^. Together, our data are consistent with the idea that in the presence of adenosine, the growth of LasR mutants is restrained by cells with an intact QS system. Although we do not understand the mechanism allowing WT constraint of LasR mutants that can grow by themselves on adenosine, it is unlikely to involve a preference for adenosine over casein as a carbon source. In our experiments, both the parent and the LasR mutant have a similar ability to use adenosine as a carbon and energy source (Fig. 5c).

## Discussion

In this study, we examined how a *P*. *aeruginosa* QS-null cheater, which does not produce the public good extracellular protease, can invade a population of cooperating cells from low frequency. To do this, we co-cultured LasR mutants and WT PAO1 in casein broth, as growth in this medium requires the QS-regulated secreted protease elastase. In contrast to most published experiments, which determined cheater fractions in endpoint experiments, we measured the change in cheater frequency over time. Our data show that the fitness advantage of LasR mutants over WT was largely manifested during early and mid-logarithmic growth, as evidenced by their faster growth rate (Fig. 1b,e) and the corresponding increase in population frequency (Fig. 1c,f). This observation is consistent with previous studies showing that LasR mutants benefit from shared resources, such as elastase, produced by WT cells, without paying the metabolic cost of producing those resources^14,15^. As previously reported, LasR mutants were also more resistant than WT to alkaline stress and cell lysis in late stationary phase via an unidentified mechanism^24^, which resulted in further increase of LasR mutant frequencies during prolonged incubation. This finding is a reminder that endpoint experiments with different incubation times or with mutants that have different growth rates can result in different outcomes. We also note that prior to our experiments, it was not known whether cheater invasion would be robust early in growth. During this period, cells have access to carry-over protease and proteolytic products in the inocula. These products could limit the relative fitness advantage of cheaters early in growth.

We also examined the effect of an adaptive mutation in *psdR* on LasR mutant invasion of a cooperating population cultured in casein broth. This is a case in point where a study is done with a fast-growing mutant. Deletion of *psdR* from both cooperators and cheaters did not affect the overall dynamics of LasR mutant invasion from low initial frequency. Our results are consistent with an earlier study, which showed that when a loss-of-function mutation in *psdR* was present in both strains, the fitness advantage of the LasR mutant was the same as when neither strain had the mutation^20^. In agreement with that study, deletion of *psdR* increased the absolute fitness of the population, which was reflected in faster growth rates of both strains (Fig. 2a,b). Our data point to the fact that the daily transfer protocol commonly used in *P*. *aeruginosa* evolution experiments in casein broth is not expected to introduce significant biases. Once mutations in *psdR* spread through the population early in the experiment, the cultures enter stationary phase within 24 h, and cheater frequency is stabilized by the time of transfer.

Because QS-null strains have a fitness advantage over cooperating cells under some conditions, we are interested in understanding how QS-regulated cooperative behavior is stabilized in populations. We have previously reported that the addition of adenosine to casein constrains LasR mutant emergence during *P*. *aeruginosa* evolution experiments^16^. Adenosine catabolism is dependent on the LasR-regulated transcription of *nuh*, which encodes a cellular nucleosidase^28^. We proposed that QS co-regulation of private goods, along with public goods, stabilizes cooperative behavior through metabolic constraint^5,16^. This interpretation has been questioned^25^. It was suggested that in our experiments, the cells evolved to grow preferentially and non-socially on adenosine even though casein was present in the medium. Consistent with our earlier work, we observed that the addition of adenosine to casein broth strongly constrained LasR mutants (Fig. 3c,d). We show that when adenosine was present in casein broth, LasR mutants did not invade the population in logarithmic phase and died more rapidly than the WT in stationary phase (Fig. 3). We do not know the mechanism for this selective death of LasR mutants, but it suggests involvement of another mechanism in addition to metabolic constraint. Our data also suggest that the benefit of private and public good co-regulation is not a consequence of carbon-source switching in stationary phase: when adenosine is present in casein broth, neither WT PAO1 nor the LasR mutant demonstrate initiation of enhanced growth at later time points in comparison to growth in casein broth without adenosine (Figs 3 and 4).

WT PAO1 does not grow readily on adenosine as a carbon source, but it can adapt to do so via amplification of the genomic region that includes *nuh* and neighboring genes^27^. We asked whether an adenosine-adapted variant would be more effective at constraining LasR mutants when adenosine was present in casein broth. Intriguingly, even though a LasR mutant of the adapted variant grew on pure adenosine as well as the parent strain (Fig. 5c), its growth was strongly constrained when it was co-cultured with the parent strain on adenosine alone (Fig. 5b) or casein plus adenosine (Fig. 4c,d). Furthermore, the adenosine-adapted LasR mutant was constrained in the co-culture with the parent strain at all stages of growth when adenosine was added to the casein medium (Fig. 4). This result indicates that the adenosine-adapted variant is more effective than WT PAO1 at outcompeting the LasR mutant when adenosine is present in the medium, even though the LasR mutant appears to catabolize adenosine as efficiently as the parent on its own.

In summary, our results are consistent with the hypothesis that QS regulation of both public and private goods plays an important role in stabilizing cooperative behavior. We have shown that *P*. *aeruginosa* strains with an intact QS system have a fitness advantage over QS-null mutants during growth in casein broth that contains adenosine, in contrast to pure casein broth. This advantage is even stronger for a strain that is adapted to grow on adenosine. Our data indicate that cheater constraint during growth in casein broth containing adenosine cannot be explained by the preferential use of adenosine over casein as a primary carbon source. A peculiar finding in our experiments is that the presence of adenosine in the medium leads to increased LasR mutant cell death when it is co-cultured with cells that have an intact QS system. Understanding the mechanism for this phenomenon was beyond the scope of this study. Stabilization of cooperative behavior in the presence of adenosine or other nucleosides may be relevant under conditions that lead to extensive cell damage, such as during infection. QS regulation of genes encoding other private enzymes potentially helps stabilize QS across multiple conditions, thus contributing to the preservation of cooperation in *P*. *aeruginosa*.

## Methods

### Bacterial strains, plasmids and culturing conditions

Bacterial strains and plasmids are listed in Table 1. Bacteria were grown in Lennox Lysogeny Broth buffered with 50 mM 3-(N-morpholino)-pro-panesulfonic acid, pH = 7.0 (LB-MOPS) or photosynthetic medium (PM)^29^ containing casein (casein broth), adenosine (adenosine broth), or both, as the sole sources of carbon and energy. All experiments were performed using a minimum of three biological replicates.

**Table 1:** Bacterial strains and plasmids used in this study.

Strain or plasmid	Relevant properties	Reference or origin
*P. aeruginosa*		
PAO1	Wild-type	^32^
PAO1-*lasR*	PAO1 containing an unmarked, in-frame *lasR* deletion	^33^
PAO1-*psdR*	PAO1 containing an unmarked, in-frame *psdR* deletion	^34^
PAO1-*psdRlasR*	PAO1-psdR containing an unmarked, in-frame *lasR* deletion	This work
PAO1-variant D	Adenosine growth variant containing gene amplification of the sequence 162685–175070	^27^
PAO1-variant D-*lasR*	PAO1-variant D containing an unmarked, in-frame *lasR* deletion	This work
PAO1-mcherry	chromosomal insertion: attTn7:: P_tac_-mCherry	This work
PAO1-*lasR*-GFP	Gm^r^; chromosomal insertion: attTn7:: P_tac_-GFP	This work
PAO1-*psdR*-GFP	chromosomal insertion: attTn7:: P_tac_-GFP	This work
PAO1-*psdRlasR*-mcherry	Gm^r^; chromosomal insertion: attTn7:: P_tac_-mCherry	This work
PAO1-variant D-mcherry	chromosomal insertion: attTn7:: P_tac_-mCherry	This work
PAO1-variant D-*lasR*-GFP	Gm^r^; chromosomal insertion: attTn7:: P_tac_-GFP	This work
*Escherichia coil*		
S17-1	*recA* pro *hsdR* RP4-2Tc::Mu-Km::Tn7	^35^
NEB5α	*fhuA2 Δ(argF-lacZ)U169 phoA glnV44 Φ80 Δ(lacZ)M15 gyrA96 recA1 relA1 endA1 thi-1 hsdR17*	NEB
*Plasmids*		
pUC18-mini-Tn7-Gm-GFP	Used for the integration of *gfp* at the *att* site	^31^
pUC18-mini-Tn7-Gm-mCherry	Derived from pUC18-mini-Tn7T-Gm^31^; used for the integration of *mCherry* at the *att* site	^36^
*pPEXG2-lasR*	pEXG2 containing sequences for *lasR* knockout	^33^
pFLP2	*flp* recombinase gene for excising a resistance marker from the fluorescent protein cassettes delivered in the pUC18-mini-Tn7T-Gm plasmids	^37^

### Strain construction

To generate mutants, we used a homologous recombination-based two-step allelic exchange approach, as described previously^30^. Briefly, *E*. *coil* S17-1 was used to deliver the plasmid to *P*. *aeruginosa* via conjugation. Merodiploids were selected on *Pseudomonas* isolation agar containing 100 µg/ml gentamicin, and deletion mutants were selected on LB agar containing 10% (w/v) sucrose and no sodium chloride. Mutant construction was confirmed by Sanger sequencing of the PCR amplicon spanning the region around the deletion. All strains used in competition experiments had a constitutively expressed *gfp* or *mCherry* at the *att* site, which we integrated using the mini Tn7 insertion system, as previously described^31^. In the LasR mutants we did not excise the gentamycin resistance marker from the fluorescent protein expression cassette.

### Competition experiments

Strains were co-cultured in casein broth or casein broth with adenosine. Overnight cultures of individual strains grown in LB-MOPS were used to inoculate fresh media to a combined optical density at 600 nm (OD_600_) of 0.05. Cultures were incubated at 37**°**C, shaking at 250 RPM, for 24 or 60 h, as indicated. To determine colony forming units (CFU) of LasR mutants and of the total population, we plated serial dilutions of the cultures on LB agar with and without 5 µg/ml gentamicin, respectively, and incubated the plates overnight at 37**°**C. To determine CFU of the strain in competition with the LasR mutant, we subtracted LasR mutant CFU from the total population CFU. To calculate the rate of change in cell density at a given time point, we used the following formula:

$${R}_{n}=\frac{\mathrm{log}({D}_{n})-\,\mathrm{log}({D}_{n-1})}{({t}_{n}-{t}_{n-1})}$$

where *R*_*n*_ is the rate of change in cell density at time point *n*, *D* is the cell density and *t* is time. pH of the cultures was determined using a pH meter. For statistical analysis, we compared rates of change in cell density using a multiple-comparisons t-test, with correction for multiple comparisons. Statistical significance was determined using the Holm-Sidak method, with alpha = 5.0%
